# Vaccination

**Published:** 1890-03-15

**Authors:** 


### VACCINATION.

Dr. Stephen C. Martin writes to the *Boston Medical and Surgical Journal* on "a pregnant cause of failure in vaccination." Instructions regarding vaccination require the arm of the vaccinee to be washed, also the instrument to be used, and the hands of the vaccinator, in a solution of carbolic acid. Afterwards corrosive sublimate was to be substituted for carbolic acid, "as being the latest favourite among germicides." But the introduction of pathogenic germs into the system is precisely the end aimed at by vaccination. Is it proper to saturate the soil in which these germs are to be sown, and everything coming in contact with them, with the most powerful germicide known? "The wound may heal admirably, but there will be no vaccination." Dr. Martin therefore recommends washing with plain soap and water, and holding the lancet for a moment in a flame which will thoroughly sterilize it. "But in the name of all that is rational do not use a drop of any fluid germicide as an adjunct."

